# A pilot study of multimodal MRI for the preoperative assessment of triple-negative breast cancer

**DOI:** 10.3389/fonc.2026.1714620

**Published:** 2026-02-04

**Authors:** Shan Luo, Rong Lin, Hui Lin, Zejuan Zhan, Xiaomin Wu, Langlang Tang, Xionghua Huang, Yuanying Liu, Pengfei Li, Junye Yao, Peng Wu, Xueyan Liao

**Affiliations:** 1 Department of Radiology, Longyan First Affiliated Hospital of Fujian Medical University, Longyan, Fujian, China; 2 Department of Pathology, Longyan First Affiliated Hospital of Fujian Medical University, Longyan, Fujian, China; 3 Department of Breast Surgery, Longyan First Affiliated Hospital of Fujian Medical University, Longyan, Fujian, China; 4 Clinical and Technical Support, Philips Healthcare, Shanghai, China

**Keywords:** diffusion-weighted imaging, dynamic contrast enhancement, magnetic resonance imaging, quantitative mapping, triple-negative breast cancer

## Abstract

**Objectives:**

To evaluate the diagnostic value of multimodal MRI by comprising quantitative mapping, diffusion-weighted imaging (DWI), and dynamic contrast-enhanced MRI (DCE-MRI) in differentiating triple-negative breast cancer (TNBC) from non-TNBC, and to investigate the correlation between significant quantitative mapping parameters and the Ki-67 proliferation index.

**Methods:**

This prospective study enrolled 120 patients with invasive breast cancer (30 TNBC and 90 non-TNBC) who underwent preoperative quantitative mapping, DWI, and DCE-MRI. DCE-MRI parameters (RE, ME, MRE, T0, TTP, WI, WO, BOE), DWI metrics (ADCmin, ADCmean, rADCmin, rADCmean), and quantitative mapping parameters (Native T1, Enhanced T1, ΔT1, ΔT1%, T2) were compared between groups. Diagnostic performance was evaluated using receiver operating characteristic (ROC) analysis and area under the curve (AUC) for both individual significant parameters and a combined predictive model. Pearson’ s correlation coefficient was used to analyze the relationship between quantitative mapping parameters and the Ki-67 index.

**Results:**

Compared to the non-TNBC group, TNBC lesions exhibited significantly lower RE, ME, WI, WO, and ΔT1% (*p* < 0.05), but higher Enhanced T1 and T2 values (*p* < 0.05). The combined model integrating DCE-MRI and quantitative mapping achieved the highest diagnostic accuracy (AUC = 0.977), outperforming DCE-MRI alone (AUC = 0.951) and quantitative mapping alone (AUC = 0.864), with sensitivity and specificity of 93.3% and 92.2%, respectively. In TNBC, the Ki-67 index showed a weak positive correlation with Enhanced T1 (r = 0.387, *p* < 0.05) and T2 (r = 0.381, *p* < 0.05).

**Conclusion:**

Parameters including RE, ME, WI, WO, Enhanced T1, ΔT1%, and T2 serve as valuable imaging biomarkers for the preoperative identification of TNBC. Integrating quantitative mapping with DCE-MRI significantly enhances diagnostic performance, offering a robust approach for differentiating TNBC from non-TNBC subtypes.

## 1 Introduction

Breast cancer remains the most prevalent malignancy among women in China, with mortality rates ranking fourth, posing a significant public health threat (1). Due to high biological heterogeneity, breast cancer exhibits considerable variation in clinical management, treatment response, and overall survival across diverse molecular subtypes (2, 3). Triple-negative breast cancer (TNBC), characterized by the absence of estrogen receptor (ER), progesterone receptor (PR), and human epidermal growth factor receptor 2 (HER2) expression, represents the most aggressive subtype, accounting for 15% to 20% of all breast cancer cases in China (4). Globally, TNBC constitutes approximately 10% to 15% of all breast cancer diagnoses (5). Recent epidemiological analyses have further highlighted significant racial and regional disparities in TNBC incidence, particularly within the United States where specific demographic cohorts and geographical regions exhibit distinct prevalence patterns (5). Given the lack of targeted therapeutic options, early and accurate diagnosis is crucial because patients with TNBC do not benefit from endocrine therapy or HER2 targeted agents and rely predominantly on chemotherapy (2, 6). Currently, immunohistochemical assessments are primarily derived from preoperative biopsy, which yields limited tissue samples and thus hampers the comprehensive evaluation of tumor heterogeneity (7).

Conventional magnetic resonance imaging (MRI) relies on subjective interpretation of morphological features such as tumor shape, signal intensity, and enhancement patterns, which limits objectivity. With the increasing demand for precision oncology, there is a growing need for advanced, quantitative imaging modalities capable of providing reliable biomarkers for objective clinical assessment. Diffusion-weighted imaging (DWI) is a non-invasive functional MRI technique that evaluates tissue microstructure by measuring the restricted diffusion of water molecules, quantified through the apparent diffusion coefficient (ADC). Dynamic contrast-enhanced MRI (DCE-MRI) assesses tumor microvascular architecture and perfusion by tracking the dynamic uptake and washout of contrast agents, typically analyzed using pharmacokinetic models. Both techniques have been widely applied in breast cancer for diagnosis, differential diagnosis, molecular subtyping, and monitoring response to neoadjuvant chemotherapy (8–12).

T2 mapping is an emerging quantitative MRI technique that enables voxel-wise measurement of T2 relaxation times, reflecting tissue free water content. Elevated free water content correlates with prolonged T2 values, providing insights into lesion composition (13). Furthermore, T2 mapping can generate color-coded parametric maps through pixel-by-pixel reconstruction, enhancing visualization and reducing observer bias (14). Similarly, T1 mapping allows specific assessment of longitudinal relaxation times at the voxel level (15). Native T1 refers to pre-contrast T1 values, while Enhanced T1 denotes post-contrast measurements, which are typically shortened due to gadolinium uptake. Initially developed for cardiac imaging, T1 mapping is increasingly being explored for oncological applications (16, 17). The high histological heterogeneity of breast cancer results in complex tissue compositions, necessitating more sensitive and quantitative imaging tools.

In the rapidly evolving landscape of oncology, precision medicine is being revolutionized by upgraded theragnostic strategies. For instance, multivariate nano-gold is being explored for the upgraded management of hepatocellular carcinoma (18). Similarly, harnessing the unique architecture of solid lipid nanoparticles has been proposed as a state-of-the-art strategy to combat colon cancer through improved drug delivery (19). Other emerging strategies include cabazitaxel-loaded redox-responsive nanocarriers based on tocopheryl chitosan and hyaluronic acid, designed to enhance anti-tumor efficacy (20). Furthermore, multifunctional nanosponges offer significant potential by integrating targeted drug delivery with imaging capabilities (21). These advancements in targeted therapy underscore the parallel imperative for equally precise, non-invasive diagnostic methods like multiparametric MRI in breast oncology.

Accurate noninvasive preoperative identification of TNBC is a critical step toward personalized medicine, enabling timely and appropriate therapeutic decision making. Therefore, this study aims to evaluate whether quantitative MRI techniques, including DWI, DCE-MRI, and T1/T2 mapping, can effectively capture the microstructural and physiological characteristics of TNBC. By constructing and comparing multiple diagnostic models, we seek to assess their individual and combined predictive performance in distinguishing TNBC from non-TNBC, thereby providing valuable insights for the development of personalized treatment strategies in breast cancer.

## 2 Materials and methods

### 2.1 Patients

This single-center prospective study was approved by the Institutional Review Board, and the requirement for informed consent was waived. From September 2022 to December 2024, we enrolled 245 consecutive patients with invasive breast cancer confirmed by core needle biopsy or surgical pathology. All subjects underwent preoperative multimodal MRI examinations, incorporating T1 and T2 mapping, diffusion-weighted imaging (DWI), and dynamic contrast-enhanced MRI (DCE-MRI) sequences. Exclusion criteria were as follows: (1) prior neoadjuvant chemotherapy, radiotherapy, or endocrine therapy (n = 5); (2) lesions manifesting exclusively as non-mass-like enhancement (n = 40); (3) insufficient clinical or pathological data (n = 46); (4) poor image quality or severe motion artifacts (n = 12); (5) neoplasms measuring less than 1 cm in the longest axis (n = 10); and (6) multifocal or multicentric tumors (n = 12). Consequently, a total of 120 patients with 120 lesions were included in the final analysis.

The sample size for this pilot study was determined based on feasibility and precedent from similar exploratory radiomics studies in breast cancer (9). A *post-hoc* power analysis (α = 0.05, β = 0.20) indicated that with 30 TNBC and 90 non-TNBC cases, our study possessed sufficient statistical power (power > 80%) to detect effect sizes (Cohen’s d) of 0.8 or greater for key MRI parameters, such as relative enhancement, maximum enhancement, and Enhanced T1 values. This represents a clinically relevant difference in this exploratory context.

### 2.2 Materials, equipment, and methods

#### 2.2.1 Image acquisition

All breast MRI examinations were performed on a 3.0-T superconducting MR scanner (Ingenia 3.0 T, Philips Healthcare, Best, The Netherlands) with patients placed in the prone position. Both breasts were positioned within a dedicated 16-channel phased-array breast surface coil. The contrast medium employed was gadobenate dimeglumine (MultiHance, Bracco, Milan, Italy).

The comprehensive imaging protocol included the following sequences: T1-weighted imaging [repetition time (TR) = 584 ms, echo time (TE) = 8 ms, matrix 296 × 292, slice thickness 4 mm, number of excitations (NEX) = 1.5]; T2-weighted spectral adiabatic inversion recovery fat-suppressed imaging (TR = 4000 ms, TE = 70 ms, matrix 312 × 255, slice thickness 4 mm, NEX = 2); diffusion-weighted imaging (DWI) [TR = 6391 ms, TE = 77 ms, matrix 128 × 111, slice thickness 4 mm, NEX = 2, 2 b-values (0 and 800 s/mm²)] (22); native T1 mapping using a Look-Locker sequence (TR = 2.4 ms, TE = 1.13 ms, matrix 144 × 136, slice thickness 6 mm, NEX = 1); T2 mapping (TR = 1000 ms, TE = 9.8 ms, matrix 108 × 97, slice thickness 6 mm, NEX = 1); and dynamic contrast-enhanced MRI (DCE-MRI) [TR = 4.4 ms, TE = 2.1 ms, matrix 220 × 313, slice thickness 1.4 mm, NEX = 1]. This sequence comprised 8 dynamic acquisitions with a total duration of 6 minutes and 33 seconds, including one pre-contrast and seven post-contrast phases. Gadobenate dimeglumine was administered intravenously at a dose of 0.2 mmol/kg and an injection rate of 2.5 mL/s, followed by a 20 mL saline flush. Image acquisition for the dynamic series was initiated simultaneously with the start of the contrast agent injection. The temporal resolution for each dynamic phase was approximately 49 seconds. A total of 8 dynamic phases were acquired over a duration of 6 minutes and 33 seconds (23).

The quantitative consistency of T1 and T2 mapping was ensured by the manufacturer’s integrated calibration algorithm, with the system undergoing regular quality control using standardized phantoms. During DCE-MRI acquisition, patients were instructed to breathe freely and smoothly. No breath-hold maneuvers were performed, as the prone position effectively minimizes respiratory motion artifacts.

#### 2.2.2 Image analyses and data processing

Two expert radiologists with 10 and 15 years of breast imaging experience analyzed all cases independently while remaining unaware of pathological findings. To evaluate measurement reproducibility, inter-observer agreement was assessed using intraclass correlation coefficients (ICC) in a randomly selected subset of 30 cases. The ICC values indicated excellent agreement for ADCmin (0.989), ME (0.979), ADCmean (0.977), Enhanced T1 (0.953), and RE (0.916), and good agreement for T2 (0.891) and Native T1 (0.881) (**Supplementary Figure S1**). Any disagreements in morphological assessments were settled via consensus-based discussion. Semi-quantitative and quantitative data were determined by calculating the mean values of the measurements obtained by both radiologists.

Morphological characteristics were analyzed based on the 2013 guidelines of Breast Imaging Reporting and Data System established by the American College of Radiology. The assessment includes the following: (1) mass features, including shape and spiculation; (2) mass enhancement patterns; (3) surrounding features, such as foci, peripheral vascularity, axillary lymph node, and alterations in the skin and nipple.

ADC value measurement: The ADC image was automatically generated upon transmission of the original DWI image to the Philips IntelliSpace Portal (ISP) workstation. Three layers exhibiting the largest radial tumor diameter, excluding regions with cystic degeneration, necrosis, and hemorrhage, were selected. A region of interest (ROI) measuring approximately 40–50 mm^2^ was positioned at the area within the tumor contour where the ADC grey level displayed the most significant visual decrease. For each tumor, a single region of interest (ROI) was delineated on the slice exhibiting the largest axial diameter. The lowest ADC value among all ROIs was designated as ADCmin. The ADC map was used to localize the tumor’s maximal cross-sectional area, where an optimally sized oval or circular ROI was carefully delineated within the lesion boundaries to calculate the ADCmean. An ROI of identical size was positioned in the corresponding area of the contralateral breast to measure the ADC value of normal breast tissue. The relative ADCmin (rADCmin; rADCmin = ADCmin/ADC of normal breast) and relative ADCmean (rADCmean; rADCmean = ADCmean/ADC of normal breast) were subsequently calculated.

DCE-MRI measurement: DCE-MRI data were processed using the Philips ISP workstation, where semi-quantitative parameters were obtained through T1-weighted perfusion analyses. The lesion size was measured in the second phase, post-enhancement within the DCE-MRI sequence. The lesion’s maximal cross-sectional width was measured in the coronal, sagittal, and axial planes, with the largest value recorded as the lesion size. Concurrently, an ROI was placed at the area of the lesion showing the most pronounced enhancement in the same phase. The semi-quantitative parameters were calculated by averaging triplicate measurements across different tissue planes. The derived metrics comprised relative enhancement (RE), maximum enhancement (ME), maximum relative enhancement (MRE), T0 time, time to peak (TTP), wash-in rate (WI), wash-out rate (WO), brevity of enhancement (BOE), and time-intensity curve (TIC). TIC patterns were categorized into three types according to enhancement dynamics: type I (inflow type), type II (plateau type), and type III (outflow type).

Quantitative mapping measurement: Native T1, Enhanced T1, and T2 mapping images were transmitted to the Philips ISP workstation to generate quantitative pseudo-color maps. Native T1, Enhanced T1, and T2 mapping values were measured within the same ROI as defined in the ADC image. Measurements were repeated across three layers and averaged to calculate ΔT1 (ΔT1 = Native T1 - Enhanced T1) and ΔT1% (ΔT1% = ΔT1/Native T1) (24–26). The synthetic MRI sequences (Look-Locker for T1, multi-echo spin-echo for T2) incorporated the manufacturer’s integrated calibration algorithm to ensure quantitative consistency across scans without requiring external phantom calibration.

#### 2.2.3 Histopathological analyses

The criteria for estrogen receptor (ER) and progesterone receptor (PR) positivity required at least 1% positively stained nuclei in 10 high-power microscopic fields (27). HER2 positivity was determined as an immunohistochemical (IHC) HER2 score of 3 or an IHC HER2 score of 2 or greater with gene amplification confirmed by fluorescence *in situ* hybridization (28). The Ki-67 index was calculated as the ratio of positively stained cells to the total cell count, assessed by evaluating 1000 cells in the area of highest staining density within the tumor. For clinical correlation analyses, a cutoff of 20% was used to define high versus low proliferation, consistent with the St. Gallen International Expert Consensus on the primary therapy of early breast cancer (29).

### 2.3 Statistical analyses

Statistical analyses were performed with MedCalc (version 22.0), SPSS (version 26.0), and Prism (version 10.4). Statistical significance was set at p < 0.05. For categorical variables, either the Chi-square test or Fisher’s exact test was applied as appropriate. Continuous data following a normal distribution were expressed as mean ± standard deviation, whereas non-normally distributed data were expressed as median (range). Differences in DWI, DCE-MRI, and quantitative mapping parameters between the TNBC and non-TNBC groups were assessed using the independent samples t-test (for normally distributed data with homogeneous variance) or the Mann-Whitney U test (for skewed distributions or uneven variance). Receiver operating characteristic (ROC) analyses were used to determine the diagnostic performance of individual parameters in distinguishing TNBC from non-TNBC. Furthermore, logistic regression was utilized to examine the predictive capability of the combined parameters. To assess the risk of overfitting and validate the combined model (DCE-MRI + quantitative mapping), we performed 5-fold cross-validation. The stability of sensitivity and specificity estimates was further evaluated using bootstrapping with 1000 resamples. Pearson correlation analyses were used to calculate the relationship between quantitative mapping parameters and the Ki-67 index.

## 3 Results

### 3.1 Clinicopathological and MRI characteristics of invasive breast cancer

A total of 120 patients (mean age, 51.4 ± 9.8 years; range, 32–76 years) were included in the final analysis. Among them, 30 patients were diagnosed with TNBC, and 90 patients were diagnosed with non-TNBC. The clinicopathological characteristics of the two groups are summarized in **Table 1**. TNBC lesions were more frequently located in the upper inner quadrant and were more likely to exhibit a round or oval shape compared to the irregular shapes often seen in non-TNBC (p < 0.05). Subgroup analysis of TNBC morphology revealed that the round or oval shape was predominant across all pathological subtypes. Specifically, 94.7% (18/19) of invasive ductal carcinomas, 100% (3/3) of invasive lobular carcinomas, and 100% (8/8) of other histological subtypes manifested as round or oval masses (**Supplementary Table S1**). Histopathologically, the TNBC group had a significantly higher proportion of Grade III tumors (66.7% vs. 36.7%, p = 0.009) and a higher Ki-67 proliferation index (68.77% ± 18.30% vs. 42.98% ± 21.05%, p < 0.001) than the non-TNBC group. No significant differences were observed in age, menopausal status, or tumor size between the two cohorts (p > 0.05), indicating comparable baseline characteristics.

**Table 1:** Comparison of clinicopathologic and MRI characteristics between TNBC and non-TNBC groups.

Parameter	TNBC (n = 30, %)	Non-TNBC (n = 90, %)	*χ^2^*/*t* value	*P* value
Age(year)	50.83 ± 9.58	52.36 ± 10.65	-0.694	0.489
Menopausal state			0.044	0.833
Yes	15(50.0)	47(52.2)		
No	15(50.0)	43(47.8)		
Maximum diameter(mm)	23.47 ± 9.56	23.30 ± 9.49	0.083	0.934
Side			1.667	0.197
Left	21(30.0)	51(56.7)		
Right	9(70.0)	39(43.3)		
Location			15.845	0.002*
Centre	0(0)	10(11.1)		
Upper outer quadrant	10(33.3)	35(38.9)		
Outer lower quadrant	2(6.7)	22(24.4)		
Upper inner quadrant	16(53.3)	17(18.9)		
Inner lower quadrant	2(6.7)	6(6.7)		
Shape			5.120	0.024*
Round/oval	29(96.7)	71(78.9)		
Irregular	1(3.3)	19(21.1)		
Spiculation			8.980	0.003*
Yes	19(63.3)	79(87.8)		
No	11(36.7)	11(12.2)		
Mass enhancement			10.055	0.005*
Homogeneity	6(20.0)	15(16.7)		
Heterogeneity	16(53.3)	70(77.8)		
Rim enhancement	8(26.7)	5(5.6)		
Time intensity curve			0.586	0.775
I	2(6.7)	4(4.4)		
II	14(46.7)	46(51.1)		
III	14(46.7)	40(44.4)		
Foci			2.236	0.135
Yes	10(33.3)	18(20.0)		
No	20(66.7)	72(80.0)		
Increased peripheral blood vessels			4.034	0.045*
Yes	28(93.3)	69(76.7)		
No	2(6.7)	21(23.3)		
Axillary fossa lymphadenectasis			0.296	0.586
Yes	10(33.3)	35(38.9)		
No	20(66.7)	55(61.1)		
Skin			1.637	0.201
Yes	4(13.3)	22(24.4)		
No	26(86.7)	68(75.6)		
Nipple			0.096	0.756
Yes	3(10.0)	13(14.4)		
No	27(90.0)	77(85.6)		
Histological grade			8.271	0.009*
1	0(0)	1(1.1)		
2	10(33.3)	56(62.2)		
3	20(66.7)	33(36.7)		
Pathological type			6.703	0.031*
Invasive ductal carcinoma	19(63.3)	76(84.4)		
Invasive lobular carcinoma	3(10.0)	2(2.2)		
Other	8(26.7)	12(13.3)		
TNM staging			1.086	0.581
I	6(20.0)	26(28.9)		
II	19(63.3)	48(53.3)		
III	5(16.7)	16(17.8)		
IV	0(0)	0(0)		
Vascular invasion			0.724	0.395
Yes	11(36.7)	41(45.6)		
No	19(63.3)	49(54.4)		
Perineural invasion			1.754	0.185
Yes	5(16.7)	26(28.9)		
No	25(83.3)	64(71.1)		
Ki-67 index(%)	68.77 ± 18.30	42.98 ± 21.05	5.994	<0.001*

Unless otherwise indicated, data are numbers of lesions, with percentages in parentheses. *p < 0.05.

### 3.2 Comparison of MRI parameters between TNBC and non-TNBC groups

**Figure 1:**
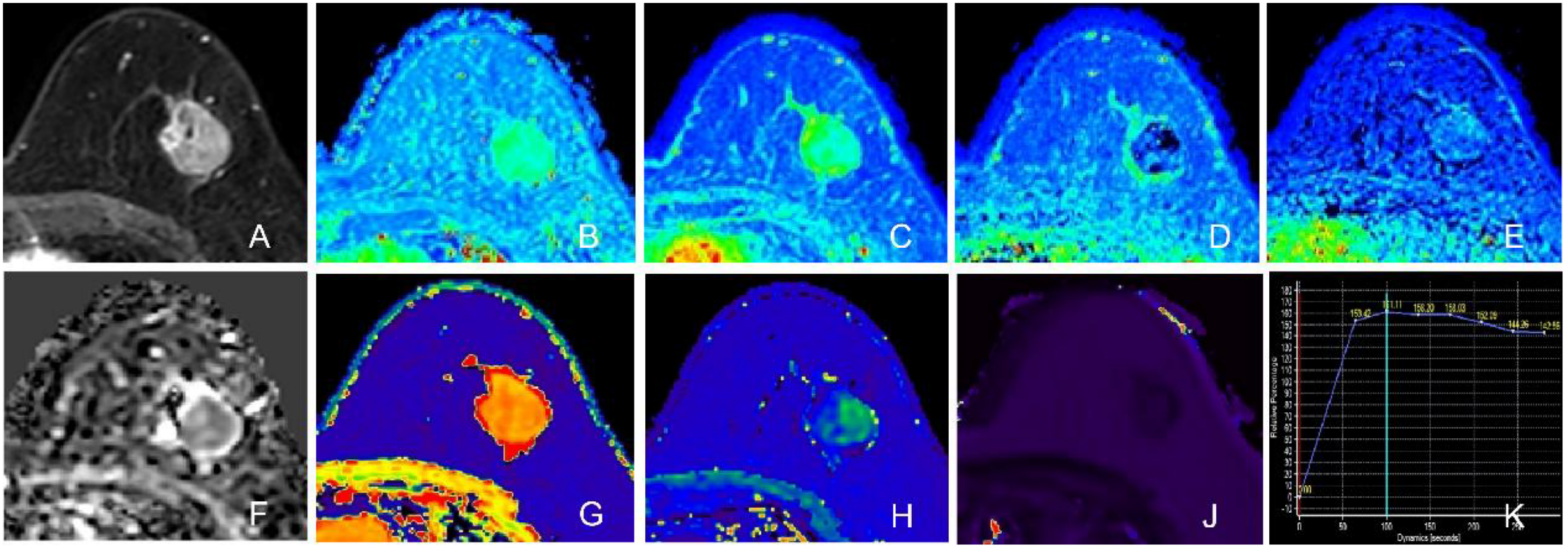
Representative images of a Triple-Negative Breast Cancer (TNBC) lesion (Invasive Ductal Carcinoma, Ki-67: 60%) in the left breast. The tumor exhibits a round shape with smooth margins and heterogeneous enhancement. **(A)** Axial DCE-MRI image. **(B)** Relative Enhancement (RE) map. **(C)** Maximum Enhancement (ME) map. **(D)** Wash-in rate (WI) map. **(E)** Wash-out rate (WO) map. **(F)** ADC map showing restricted diffusion. **(G)** Native T1 map. **(H)** Enhanced T1 map. **(J)** T2 map. **(K)** Time-Intensity Curve (TIC) showing an outflow pattern (Type III).

**Figure 2:**
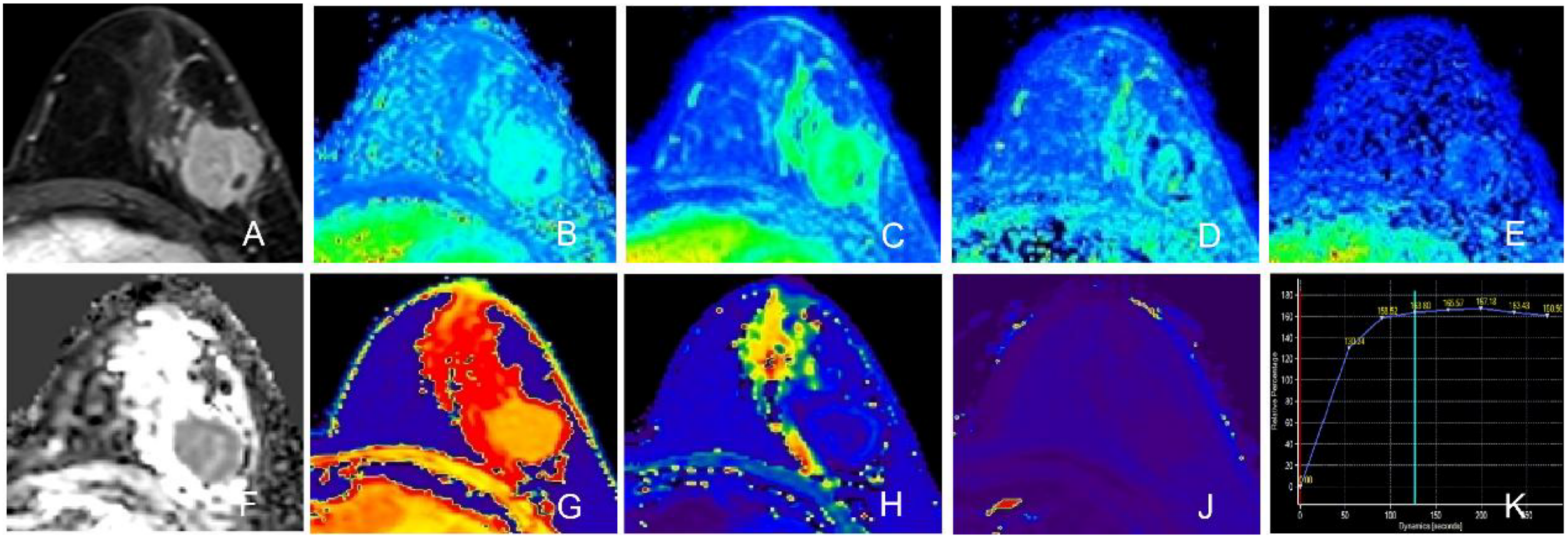
Representative images of a non-TNBC lesion (Luminal B subtype, Invasive Ductal Carcinoma, Ki-67: 30%) in the left breast. The tumor exhibits an irregular shape with spiculation and heterogeneous enhancement. **(A)** Axial DCE-MRI image. **(B)** Relative Enhancement (RE) map. **(C)** Maximum Enhancement (ME) map. **(D)** Wash-in rate (WI) map. **(E)** Wash-out rate (WO) map. **(F)** ADC map. **(G)** Native T1 map. **(H)** Enhanced T1 map. **(J)** T2 map. **(K)** Time-Intensity Curve (TIC) showing a plateau pattern (Type II).

Quantitative MRI analysis revealed significant disparities in hemodynamic and mapping parameters between the subtypes, as detailed in **Table 2** and illustrated in **Figures 1**, **2**. Regarding DCE-MRI metrics, the TNBC group exhibited significantly lower values for Relative Enhancement (RE) (169.26% ± 29.04% vs. 198.36% ± 36.33%, p < 0.001), Maximum Enhancement (ME) (183.56 ± 54.13 vs. 325.93 ± 129.49, p < 0.001), Wash-in rate (WI) (1.34 ± 0.41 s^-1^ vs. 2.33 ± 0.68 s^-1^, p < 0.001), and Wash-out rate (WO) (0.22 ± 0.11 s^-1^ vs. 0.40 ± 0.27 s^-1^, p < 0.001) compared to the non-TNBC group. In contrast, no statistically significant differences were observed in diffusion-weighted imaging parameters, including ADCmin (0.69 ± 0.17 × 10^–3^ mm^2^/s vs. 0.64 ± 0.21 × 10^–3^ mm^2^/s, p = 0.473) and ADCmean (0.93 ± 0.17 × 10^–3^ mm^2^/s vs. 0.90 ± 0.23 × 10^–3^ mm^2^/s, p = 0.423). However, quantitative mapping parameters showed marked differentiation; the TNBC group demonstrated significantly higher Enhanced T1 values (487.54 ± 75.57 ms vs. 417.09 ± 68.05 ms, p < 0.001) and T2 values (99.36 ± 13.23 ms vs. 83.33 ± 10.04 ms, p < 0.001). Furthermore, ΔT1% was significantly lower in TNBC (0.66 ± 0.05 vs. 0.70 ± 0.05, p < 0.001).

**Table 2:** Comparison of DWI, DCE-MRI and quantitative mapping parameters between TNBC and non-TNBC groups.

Sequence and parameter	TNBC (n = 30)	Non-TNBC (n = 90)	*t* value	*P* value
DCE				
RE(%)	169.26 ± 29.04	198.36 ± 36.33	-3.979	<0.001*
ME	183.56 ± 54.13	325.93 ± 129.49	-8.449	<0.001*
MRE(%)	113.06 ± 51.71	106.57 ± 55.82	0.561	0.576
T0(s)	64.62 ± 11.51	65.68 ± 13.07	-0.395	0.693
TTP(s)	174.52 ± 82.63	185.85 ± 79.95	-0.667	0.506
WI(s^-1^)	1.34 ± 0.41	2.33 ± 0.68	-9.606	<0.001*
WO(s^-1^)	0.22 ± 0.11	0.40 ± 0.27	-5.076	<0.001*
BOE(s)	119.00 ± 77.97	145.69 ± 70.44	-1.749	0.083
ADC				
ADCmin(×10^-3^mm^2^/s)	0.69 ± 0.17	0.64 ± 0.21	0.720	0.473
ADCmean(×10^-3^mm^2^/s)	0.93 ± 0.17	0.90 ± 0.23	0.804	0.423
rADCmin	0.55 ± 0.12	0.59 ± 0.23	-1.201	0.233
rADCmean	0.57 ± 0.09	0.60 ± 0.17	-0.817	0.416
T1 mapping				
Native T1(ms)	1445.37 ± 151.75	1412.23 ± 139.45	1.103	0.272
Enhanced T1(ms)	487.54 ± 75.57	417.09 ± 68.05	4.776	<0.001*
ΔT1(ms)	957.83 ± 140.95	995.14 ± 136.58	-1.286	0.201
ΔT1%	0.66 ± 0.05	0.70 ± 0.05	-4.160	<0.001*
T2 mapping				
T2(ms)	99.36 ± 13.23	83.33 ± 10.04	6.080	<0.001*

Data are presented as mean ± standard deviation. DCE-MRI Parameters: RE: Relative Enhancement (%); ME: Maximum Enhancement (arbitrary units); WI: Wash-in Rate (s^−1^); WO: Wash-out Rate (s^−1^); MRE: Maximum Relative Enhancement (%); T0: Arrival Time (s); TTP: Time to Peak (s); BOE: Brevity of Enhancement (s). DWI Parameters: ADCmin: minimum Apparent Diffusion Coefficient (×10^−3^ mm^2^/s); ADCmean: mean Apparent Diffusion Coefficient (×10^−3^ mm²/s); rADCmin: relative ADCmin (ratio of lesion ADCmin to contralateral normal tissue ADC); rADCmean: relative ADCmean (ratio of lesion ADCmean to contralateral normal tissue ADC). Quantitative Mapping Parameters: Native T1: T1 relaxation time pre-contrast (ms); Enhanced T1: T1 relaxation time post-contrast (ms); ΔT1: absolute T1 reduction (ms); ΔT1%: percentage T1 reduction (%); T2: T2 relaxation time (ms). *Statistical significance: *p < 0.05.

### 3.3 Diagnostic efficacy of DCE-MRI parameters

**Figure 3:**
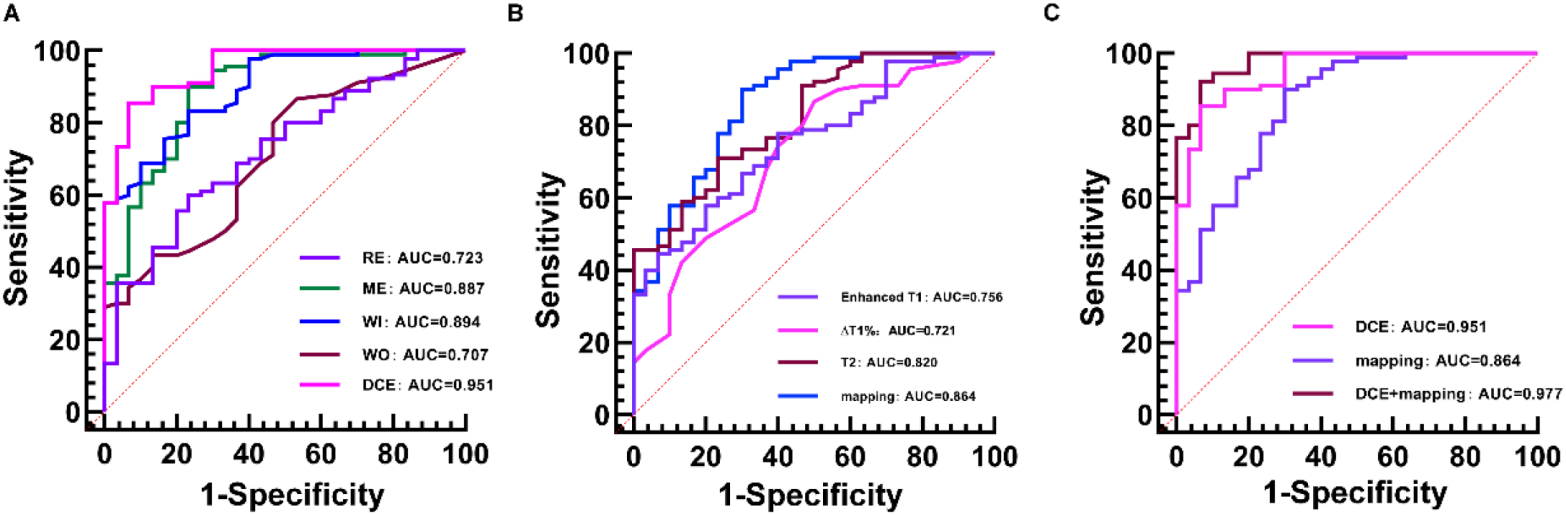
Receiver operating characteristic (ROC) curves evaluating the diagnostic performance of different MRI parameters and models for distinguishing TNBC from non-TNBC. **(A)** ROC curves of individual DCE-MRI parameters (RE, ME, WI, WO) and their combination. **(B)** ROC curves of quantitative mapping parameters (Enhanced T1, ΔT1%, T2) and their combination. **(C)** Comparison of diagnostic performance among the DCE-MRI model, Quantitative Mapping model, and the Combined Model (DCE-MRI + Quantitative Mapping). The Combined Model achieved the highest Area Under the Curve (AUC = 0.977).

To assess the discriminative power of hemodynamic features, Receiver Operating Characteristic (ROC) analyses were performed for the significant DCE-MRI parameters, as summarized in **Table 3** and **Figure 3A**. Among the individual metrics, Wash-in rate (WI) and Maximum Enhancement (ME) demonstrated robust diagnostic potential, achieving AUC values of 0.894 (95% CI: 0.825–0.943) and 0.887 (95% CI: 0.816–0.937), respectively. Relative Enhancement (RE) and Wash-out rate (WO) showed moderate diagnostic utility with AUCs of 0.723 and 0.707. Notably, the integration of these four parameters into a combined DCE-MRI model yielded superior performance, achieving an AUC of 0.951 (95% CI: 0.896–0.982) with a sensitivity of 93.3% and specificity of 85.6% (**Table 4**).

**Table 3:** Diagnostic efficacy of DCE-MRI (RE, ME, WI, WO) and quantitative mapping (Enhanced T1, ΔT1%, T2) for TNBC.

Sequence and parameter	Cutoff value	Sensitivity (%)	Specificity (%)	AUC	95% CI
DCE-MRI					
RE(%)	183.51	76.7	60.0	0.723	0.633~0.800
ME	211.15	76.7	90.0	0.887	0.816~0.937
WI(s^-1^)	1.700	76.7	83.3	0.894	0.825~0.943
WO(s^-1^)	0.165	46.7	86.7	0.707	0.617~0.787
Quantitative mapping					
Enhanced T1(ms)	420.57	80.0	57.8	0.756	0.669~0.830
ΔT1%	0.652	50.0	87.8	0.721	0.631~0.799
T2(ms)	89.47	76.7	71.1	0.820	0.739~0.884

**Table 4:** Diagnostic efficacy of DCE-MRI, quantitative mapping and combined model for TNBC.

Sequence	Sensitivity (%)	Specificity (%)	AUC	95%CI
DCE-MRI	93.3	85.6	0.951	0.896~0.982
Quantitative mapping	70.0	90.0	0.864	0.789~0.919
DCE-MRI + quantitative mapping	93.3	92.2	0.977	0.932~0.996

### 3.4 Diagnostic efficacy of quantitative mapping parameters

The diagnostic utility of quantitative mapping parameters was similarly evaluated (**Table 3**; **Figure 3B**). T2 mapping emerged as the most effective individual predictor in this category, with an AUC of 0.820 (95% CI: 0.739–0.884), followed by Enhanced T1 (AUC = 0.756) and ΔT1% (AUC = 0.721). The combined quantitative mapping model enhanced the overall specificity to 90.0% (95% CI: 81.9–95.3), although sensitivity remained moderate at 70.0% (95% CI: 50.6–85.3). The resulting AUC for the combined mapping model was 0.864 (95% CI: 0.789–0.919), indicating that while mapping parameters are highly specific, they are most powerful when used in conjunction with other modalities.

### 3.5 Comparison of diagnostic models

The overall diagnostic performance of the individual and combined models is presented in **Table 4** and **Figure 3C**. The integrated model, which combined both DCE-MRI and quantitative mapping parameters, achieved the highest diagnostic accuracy. This comprehensive model yielded an AUC of 0.977 (95% CI: 0.932–0.996), significantly outperforming either modality alone. It demonstrated balanced and high-performance metrics, with a sensitivity of 93.3% (95% CI: 77.9–99.2) and a specificity of 92.2% (95% CI: 84.6–96.8). To validate the robustness and generalizability of these findings, a 5-fold cross-validation was performed, which resulted in a mean AUC of 0.962 (SD: 0.015), confirming the stability of the combined diagnostic model.

### 3.6 Correlation with Ki-67 index

**Figure 4:**
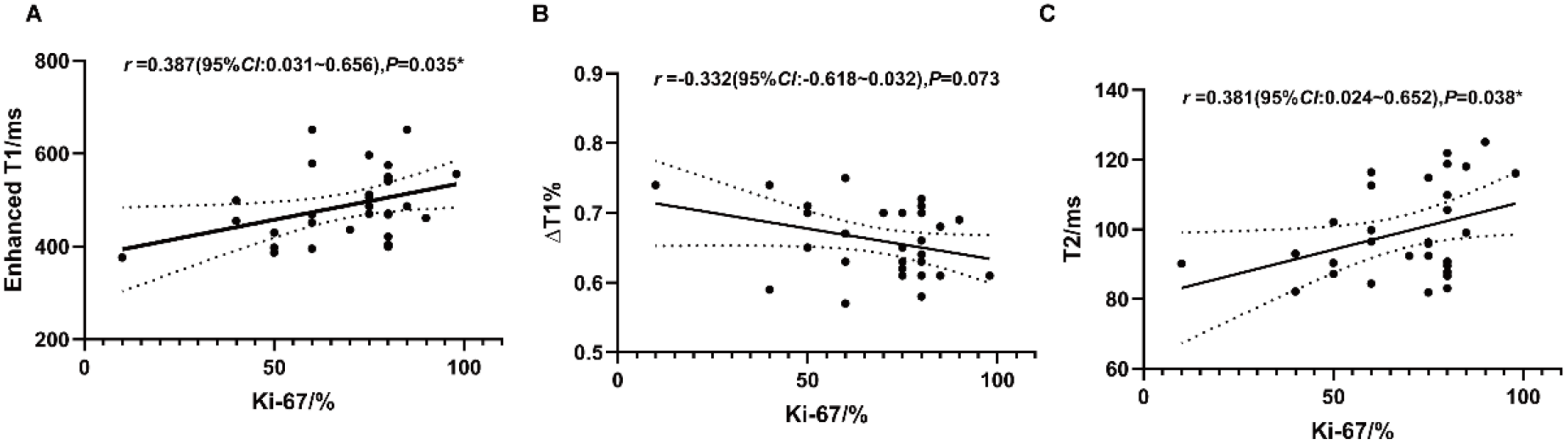
Scatter plots illustrating the correlation between quantitative mapping parameters and the Ki-67 proliferation index in the TNBC group. **(A)** Correlation between Enhanced T1 values and Ki-67 index. **(B)** Correlation between ΔT1% and Ki-67 index. **(C)** Correlation between T2 values and Ki-67 index. Each dot represents an individual tumor lesion. Pearson’s correlation coefficients (r) and p-values are indicated in the respective plots.

The results indicated a significant positive correlation between Enhanced T1 values and the Ki-67 proliferation index (**Figure 4A**; r = 0.387, 95% CI: 0.031–0.656, p = 0.035). Notably, both Enhanced T1 and T2 values showed a progressive increasing trend concomitant with higher Ki-67 indices. In contrast, no statistically significant correlation was observed between ΔT1% and the Ki-67 index (**Figure 4B**; r = -0.332, 95% CI: -0.618–0.032, p = 0.073), suggesting that relative T1 changes may be less sensitive to proliferative activity than absolute relaxation times. Similarly, T2 values exhibited a positive correlation with Ki-67 levels (**Figure 4C**; r = 0.381, 95% CI: 0.024–0.652, p = 0.038).

## 4 Discussion

The selection of complex treatment regimens for breast cancer primarily depends on hormone receptor and HER2 expression status. Patients with hormone receptor-positive tumors are eligible for endocrine therapy, whereas those with HER2-positive tumors benefit from anti-HER2 targeted therapy. In contrast, patients whose tumors lack expression of both hormone receptors and HER2, commonly referred to as triple-negative breast cancer (TNBC), have limited therapeutic options and generally exhibit a poorer prognosis (30). Breast cancer is characterized by substantial tumor heterogeneity. Currently, molecular subtyping research largely relies on dynamic contrast-enhanced MRI (DCE-MRI), diffusion-weighted imaging (DWI), and their derived sequences (1, 22). This study innovatively incorporated novel mapping sequences into the existing framework. By systematically analyzing quantitative mapping, DCE-MRI, DWI, and integrated models, we identified RE, ME, WI, WO, Enhanced T1, ΔT1%, and T2 as significant predictive biomarkers. Furthermore, the multimodal integration of DCE-MRI with quantitative mapping demonstrated enhanced preoperative diagnostic accuracy, holding promise for guiding more precise clinical decision-making.

### 4.1 Morphological and pathological characteristics

First, we evaluated morphological characteristics on DCE-MRI. Our results demonstrated that TNBC predominantly exhibited a round or oval morphology, which was more prevalent than in non-TNBC. Concurrently, the incidence of spiculation in TNBC was significantly lower, consistent with prior studies (31, 32). This suggests that TNBC often mimics the imaging features of benign breast tumors, posing challenges for accurate preoperative diagnosis. Previous studies have indicated that spiculation is associated with the expression of estrogen receptor (ER) and progesterone receptor (PR) genes (33–35). This implies that the absence of ER and PR expression contributes to the smooth tumor margins typical of TNBC. Moreover, TNBC has been reported to lack a pronounced fibrotic connective tissue response (32). The reduced occurrence of spiculation in the TNBC group may be attributed to limited interstitial reaction and diminished fibrous connective tissue proliferation resulting from cancer cell infiltration.

Heterogeneous enhancement and necrosis were more frequently observed in TNBC, with a higher proportion of rim enhancement compared to non-TNBC. Additionally, increased peritumoral vascularity was noted, aligning with previous findings of elevated microvascular density at the tumor periphery. This phenomenon is likely driven by rapid cancer cell proliferation leading to central fibrosis and necrosis (36–38). This hypothesis is further supported by the significantly higher Ki-67 index observed in the TNBC group in our cohort. Pathologically, the smooth, non-spiculated tumor margins are consistent with a reduced desmoplastic reaction, associated with ER/PR negativity and a pushing, expansive growth pattern driven by high proliferative activity. Thus, the round or oval shape with minimal spiculation represents a radiological manifestation of TNBC’s distinct pathobiological behavior.

### 4.2 Hemodynamic features and paradoxical perfusion

Beyond morphological characteristics, we evaluated semi-quantitative hemodynamic parameters derived from DCE-MRI. No significant intergroup difference was observed in time-intensity curve (TIC) type classification (p > 0.05). Currently, there is no consensus regarding the typical TIC pattern of TNBC, and existing studies indicate that differences in TIC types often lack statistical significance (22, 39, 40). Semi-quantitative DCE-MRI parameters provide a more accurate assessment of intratumoral microvascular density and vascular permeability, offering insights beyond TIC classification alone (41, 42).

Tumor aggressiveness is generally positively correlated with intratumoral hemodynamics. High-grade malignancies typically exhibit increased perfusion and vascular permeability, leading to elevated peak enhancement and rapid wash-in. Contrary to expectation, our study found that TNBC lesions showed significantly lower values for semi-quantitative parameters (RE, ME, WI, WO). This seemingly paradoxical finding may point to a distinct tumor microenvironment in TNBC. We propose that this pattern stems from an immature, hyperpermeable, and architecturally disorganized vasculature. Such vessels facilitate rapid extravasation of contrast agent into the interstitial space and equally rapid clearance, preventing the accumulation necessary to reach high enhancement peaks during standard acquisition. This interpretation aligns with evidence that TNBCs often display elevated quantitative perfusion parameters, such as K^trans^, which mark hyperpermeable yet functionally inefficient vasculature (43, 44). Furthermore, the lack of HER2 expression in TNBC has been hypothesized to impair local vascular endothelial growth factor (VEGF) production, potentially contributing to this abnormal vascular phenotype (43, 45).

### 4.3 Diagnostic value and limitations of diffusion-weighted imaging

In contrast to the DCE-MRI findings, our DWI analysis revealed no statistically significant differences in ADC metrics between TNBC and non-TNBC groups (p > 0.05). This aligns with several previous reports (11, 46, 47). The lack of significant ADC differentiation may be attributed to TNBC’s complex microstructure, where opposing effects neutralize the net ADC value. Specifically, the characteristic high cellularity of TNBC tends to restrict water diffusion and lower ADC values (10). Conversely, the frequent presence of necrosis and edema (observed as rim enhancement) tends to increase water diffusivity. These competing factors likely cancel each other out. Furthermore, the use of a single intermediate b-value of 800 s/mm² in our protocol represents a standardized clinical compromise. While maintaining optimal signal-to-noise ratio, it may lack the sensitivity to disentangle these microstructural subtleties compared to multi-b-value protocols or advanced models like intravoxel incoherent motion (IVIM) (22).

### 4.4 Value of quantitative mapping and pathological basis

The integration of quantitative T1 and T2 mapping into a multimodal MRI protocol adds a novel, objective dimension to preoperative TNBC assessment. Our study demonstrates that the TNBC group exhibited decreased ΔT1% but increased Enhanced T1 and T2 values. Mechanistically, VEGF expression is differentially regulated by HER2 and ER status (45, 48, 49). In TNBC, the absence of both receptors may lead to relative deficiencies in VEGF-driven angiogenesis stability, resulting in comparatively poorer blood supply retention. This explains the smaller post-contrast reduction in T1 relaxation time (higher Enhanced T1, lower ΔT1%). Additionally, elevated T2 values in TNBC likely reflect increased free water content, attributable to characteristically high cellular density and frequent microscopic necrotic foci and edema (50, 51).

### 4.5 Synergistic diagnostic performance

The study demonstrated that while DCE-MRI alone achieved high sensitivity and quantitative mapping alone showed high specificity, integrating these modalities yielded substantially improved performance. The combined model maintained high sensitivity at 93.3% while elevating specificity to 92.2%. This synergistic model outperforms single-modality approaches, demonstrating significant promise in minimizing both false positive and false negative diagnoses (14, 39). This highlights its potential for clinical application in distinguishing aggressive breast cancer subtypes.

### 4.6 Correlation with proliferation marker Ki-67

Our study identified significant, albeit weak, positive correlations between the Ki-67 proliferation index and both Enhanced T1 and T2 values in TNBC. The correlation with T2 may be explained by TNBC pathophysiology, where rapid proliferation leads to a hypoxic and necrotic microenvironment, increasing tissue water content and T2 signal (51). While previous studies grouped tumors by Ki-67 expression to show relationships with T2, our analysis provides a direct quantitative assessment. However, given the modest correlation strength, mapping parameters alone are likely insufficient for precise individual Ki-67 prediction but serve as valuable supportive biomarkers (52, 53).

### 4.7 Limitations and future prospects

This study has several limitations. First, its single-center design and relatively small sample size, particularly within the TNBC subgroup, may limit generalizability. However, *post-hoc* power analysis indicated sufficient statistical power to detect significant differences in key parameters. Future multi-center studies with larger cohorts are needed for validation. Second, manual lesion delineation introduces subjectivity. Future implementation of AI for automated segmentation could improve precision (54). Third, the lack of direct spatial correlation between MRI features and histopathology limits definitive attribution. Future studies incorporating MRI-guided biopsy could enable precise radiologic-pathologic correlation. Finally, conventional mono-exponential DWI may lack sensitivity. Future work using multi-b-value acquisitions or advanced models (IVIM, DKI) could provide more nuanced characterization (10).

## 5 Conclusion

In summary, this study demonstrates that the integration of quantitative T1 and T2 mapping with conventional DCE-MRI parameters provides a highly effective and noninvasive tool for the preoperative differentiation of TNBC. The combined predictive model achieves optimal diagnostic performance by capturing the distinct pathophysiological features of TNBC, including its unique tumor microenvironment and perfusion patterns. The ability to accurately identify TNBC preoperatively has profound clinical implications. It allows for the stratification of patients who are most likely to benefit from aggressive systemic therapies, such as the prioritization of neoadjuvant chemotherapy, which is crucial for achieving pathological complete response and improving long term prognosis. While the promising results from this pilot study require validation in larger cohorts, this integrated imaging framework represents a tangible advance toward personalized, precision medicine in breast cancer management.

## Data Availability

The original contributions presented in the study are included in the article/**Supplementary Material**. Further inquiries can be directed to the corresponding author.
