# Supplementary figures and images for: A pilot study of multimodal MRI for the preoperative assessment of triple-negative breast cancer
Source: Front Oncol. 2026 Feb 4;16:1714620. doi: 10.3389/fonc.2026.1714620 (PMC12913062; doi:10.3389/fonc.2026.1714620)

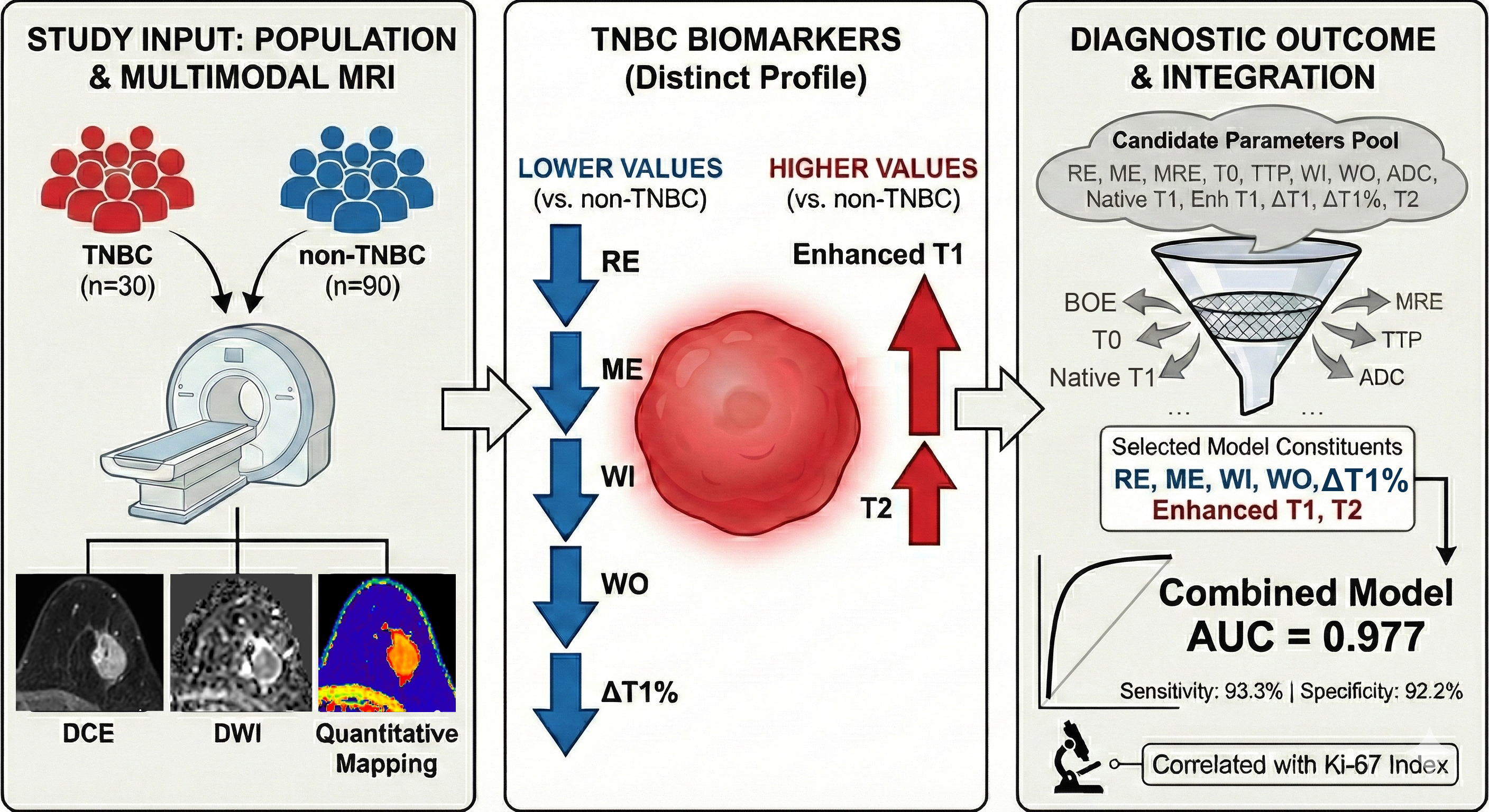

Supplement: Supplementary file 2 [file Image2.jpeg]

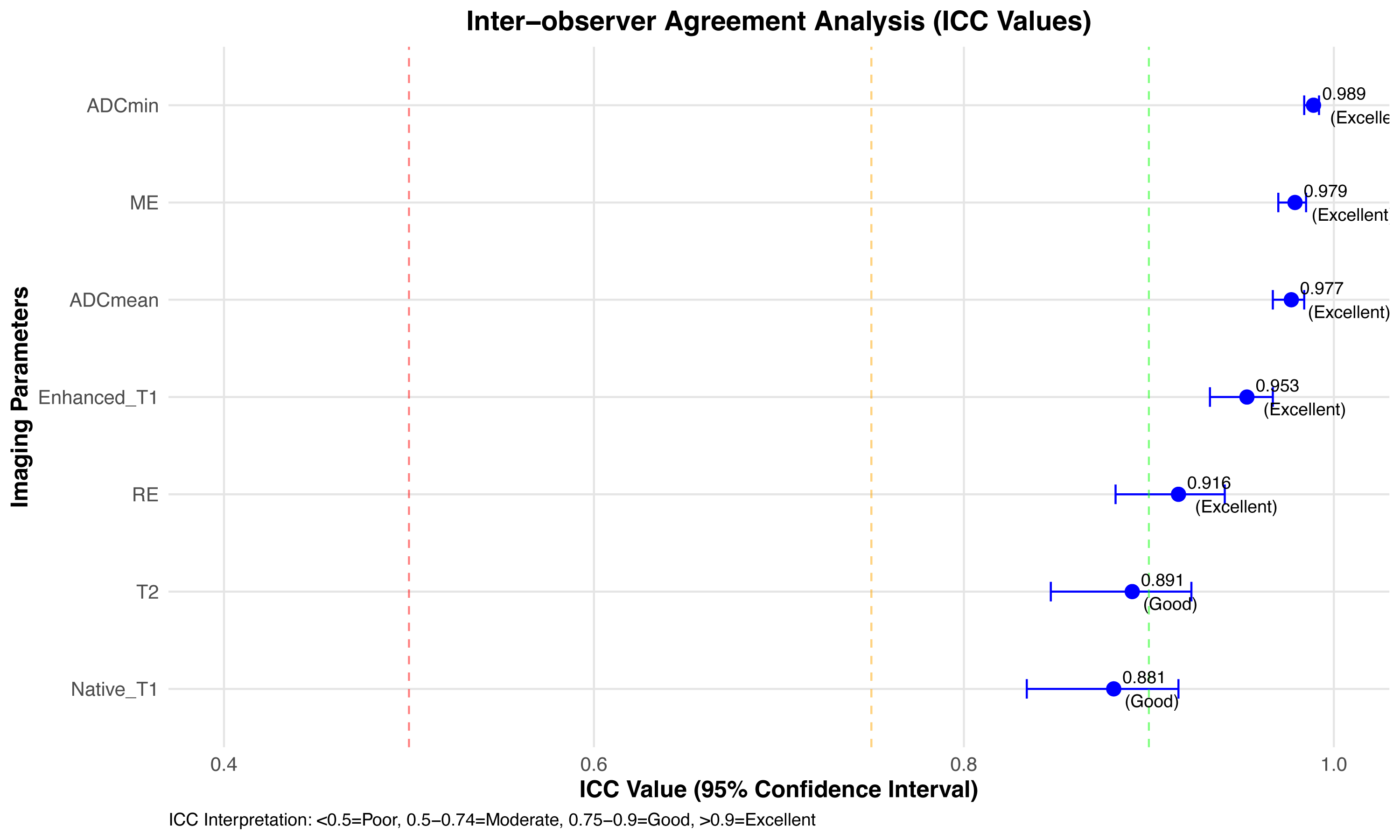

Supplement: Supplementary file 3 [file Image1.tif]
